# Tumor infiltrating lymphocytes expanded from pediatric neuroblastoma display heterogeneity of phenotype and function

**DOI:** 10.1371/journal.pone.0216373

**Published:** 2019-08-09

**Authors:** Marina Ollé Hurtado, Jolien Wolbert, Jonathan Fisher, Barry Flutter, Sian Stafford, Jack Barton, Neha Jain, Giuseppe Barone, Yvonne Majani, John Anderson

**Affiliations:** 1 Cancer Section, Developmental Biology and Cancer Programme, UCL Great Ormond Street Institute of Child Health, London, England, United Kingdom; 2 Department of Oncology, Great Ormond Street Hospital, London, England, United Kingdom; Peter MacCallum Cancer Centre, AUSTRALIA

## Abstract

Adoptive transfer of *ex vivo* expanded tumor infiltrating lymphocytes (TILs) has led to clinical benefit in some patients with melanoma but has not demonstrated convincing efficacy in other solid cancers. Whilst the presence of TILs in many types of cancer is often associated with better clinical prognosis, their function has not been systematically evaluated across cancer types. Responses to immunological checkpoint inhibitors in a wide range of cancers, including those for which adoptive transfer of expanded TILs has not shown clinical benefit, has clearly delineated a number of tumor type associated with tumor-reactive lymphocytes capable of effecting tumor remissions. Neuroblastoma is an aggressive childhood solid cancer in which immunotherapy with GD2-directed antibodies confers a proven survival advantage through incompletely understood mechanisms. We therefore evaluated the feasibility of *ex vivo* expansion of TILs from freshly resected neuroblastoma tumors and the potential therapeutic utility of TIL expansions. TILs were successfully expanded from both tumor biopsies or resections. Significant numbers of NKT and γδT cells were identified alongside the mixed population of cytotoxic (CD8^+^) and helper (CD4^+^) T cells of both effector and central memory phenotypes. Isolated TILs were broadly non-reactive against autologous tumor and neuroblastoma cell lines, so enhancement of neuroblastoma killing was attained by transducing TILs with a second-generation chimeric antigen receptor (CAR) targeting GD2. CAR-TILs demonstrated antigen-specific cytotoxicity against tumor cell lines. This study is the first to show reproducible expansion of TILs from pediatric neuroblastoma, the high proportion of innate-like lymphocytes, and the feasibility to use CAR-TILs therapeutically.

## Introduction

Tumor infiltrating lymphocytes (TILs) are found in most solid cancers but understanding of their function and purpose remains incomplete. Adult melanoma patients have been extensively evaluated for adoptive transfer of *ex vivo* expanded TILs derived from tumor tissue, by culturing single cell suspensions or tumor fragments with high doses of interleukin 2 (IL-2). Expanded TILs were able to recognize autologous tumor tissue *in vitro* [1]. In clinical trials in melanoma, TIL therapy has demonstrated response rates of 50% and higher, and durable complete response rates of 20% in heavily pre-treated patients [2]. The protocol for expanding TILs from melanoma has not been as successful when used to grow TILs from most other solid tumors [3,4]. In 2015 Baldan *et al*. published a modified protocol which used CD3/CD28 antibody stimulation and led to expansion of TILs from renal cell carcinoma [5]. Since then, isolation and expansion of tumor-reactive TILs has been successful in a wide range of cancers, such as hepatocellular carcinoma or triple-negative breast cancer [6,7], but not thus far from neuroblastoma.

Neuroblastoma is the most common non-central nervous system solid cancer affecting children. High-risk neuroblastoma remains a major clinical unmet need due to its high relapse rate [8] and very poor prognosis following recurrence [9]. The role of tumor infiltrating lymphocytes in controlling disease progression in neuroblastoma is largely unknown, although recent studies have suggested the role for CD4 T cells in immune surveillance [10] whilst downregulation of Major Histocompatibility Complex (MHC) molecules is consistent with immune evasion [11]. Nevertheless, it is still unclear whether immune-checkpoint inhibitors might be effective in unleashing naturally tumor-resident and tumor-reactive TILs to treat neuroblastoma. If the evolving paradigm described in adult cancer holds true in neuroblastoma [12,13], its low neoantigen frequency [14] might explain the lack of response to immune-checkpoint inhibitors possibly linked to the absence of tumor-reactive TILs [15].

An emerging approach to overcome the constraints of MHC-restricted T cell receptor (TCR) activation, and lack of naturally occurring anti-tumor T cell responses, is to genetically modify a patient’s T cells to express chimeric antigen receptors (CARs) [16]. CARs are engineered synthetic receptors that can be genetically transferred into T cells to confer a new specific reactivity typically defined by the variable regions of an antibody [17]. CAR T-based immunotherapy has demonstrated encouraging efficacy against lymphoid malignancies for example CD19 CAR T cells therapy has a remarkable 85% complete response rates averaged across several clinical trials [18–20]. In contrast, the solid cancer environment appears to represent a significant barrier to survival, expansion and persistence of CAR-T cells [21]. GD2 is a disialoganglioside highly expressed on tumors of neuroectodermal origin, such as neuroblastoma, melanoma, or retinoblastoma, but with low expression on normal tissues [22]. CAR T cell therapy for neuroblastoma is being explored and several clinical studies are ongoing to test the infusion of transduced cells in children with relapsed or refractory disease [23,24].

Because of previous studies indicating the neuroblastoma microenvironment might be permissive to tumor-reactive lymphocytes [25], the current study depicts the role of TILs in neuroblastoma by evaluating their phenotype and function following *ex vivo* expansion. We demonstrate that the expanded TILs contain a high proportion of atypical lymphocytes and show little evidence of reactivity against autologous tumor, although they demonstrate killing of allogeneic neuroblastoma cell lines. Expanded TILs could be transduced with a GD2-specific second-generation CAR leading to enhanced neuroblastoma cell cytotoxicity. A hypothesis to be tested is that CAR-TIL might have greater natural tropism for tumor than blood borne counterparts.

## Results

### Consistent expansion of TILs from neuroblastoma tissue to clinically relevant cell numbers

**Fig 1:**
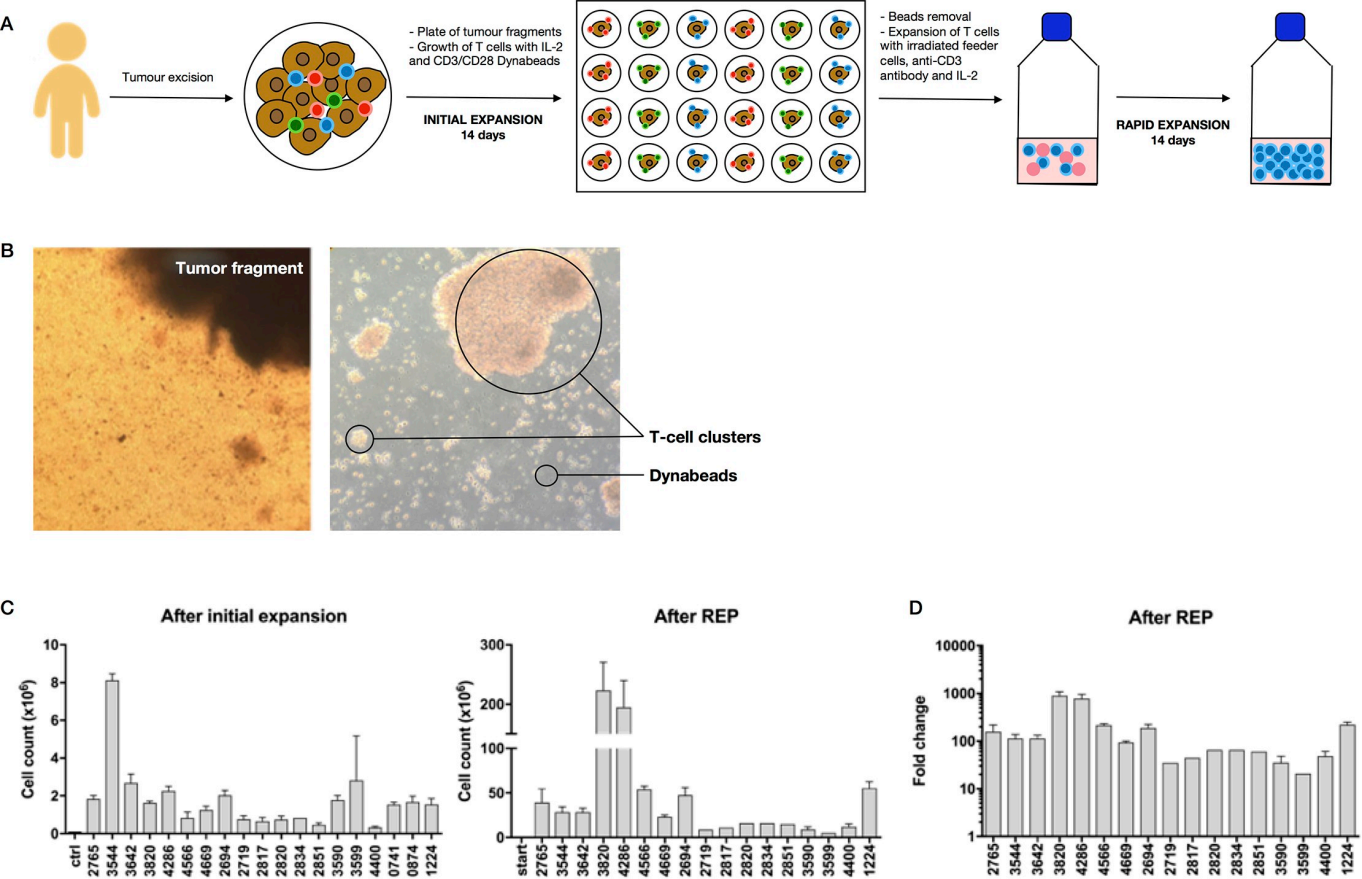
Large numbers of TILs can be expanded from neuroblastoma samples. (A) Overview of TILs expansion protocol. (B) Representative microscope images of TILs at day 14 of initial expansion (left), and TILs at the end of REP (right). (C) Cell count per well at the end of initial expansion (left) and cell count per 75cm^2^ flask at the end of REP (right). D) Fold change after REP. Data represented as mean ± Standard Error of Mean (SEM). Each case comprised a mean of 7.3 fragments (range 1–30 n = 19) for initial expansion and mean of 3 (range 1–8 n = 17) independent cultures per case for REP. Note case 1224 is the average of 4 independent tumors from the same patient. Accurate cell counts were missing from 4 further cases that went on to REP.

Over a 30-month period, 30 samples from 24 pediatric neuroblastoma patients were received. We did not attempt expansion of TILs in IL-2 alone but rather initial expansion was initiated for all the samples using 3000u/ml IL-2 in combination with CD3/CD28 beads (Fig 1A). Around the fourth day of culture, TILs start to migrate out of the tumor tissue to form a confluent layer and cluster with the CD3/CD28 beads around the tumor fragments (Fig 1B, left). After 14 days of initial expansion, TILs were effectively expanded from 23/30 samples, and were immunophenotyped in 16 cases. Although tumor-related variability in the number of TILs expanded was observed, as much as 8 x106 TILs per well could be obtained after 2 weeks expansion (Fig 1C, left). Control cultures containing tumor fragments but without IL-2 or CD3/CD28 Dynabeads showed no expansion of TILs.

To further expand TILs and reach clinically relevant numbers, 0.25x106 TILs from each primary expansion were put into Rapid Expansion Protocol (REP) with 1000u/ml IL-2, irradiated PBMCs as feeder cells, and CD3 antibody. Around the sixth day of REP, clusters of T cells were observed under the microscope (Fig 1B, right). There was successful expansion in 21/23 cases put into REP and total cell numbers of over 200 x10^6^ TILs could be obtained per 75cm^2^ flask (representing one initial well/tissue fragment) at the end of the REP (Fig 1C, right). During REP there was a mean fold increase of 296.59±52.22 fold (Fig 1D). Multiplying by the number of fragments per case put into initial expansion it is possible to estimate the total number of TILs that could be attained per small tumor fragment in a clinical protocol. This mean extrapolated final cell product is 541.22±95.26x106.

Surgical or biopsy sample sources were equally successful for both initial TILs expansion (73% vs 79%) and rapid TILs expansion (73% vs 68%). There were also no significant differences in the TILs expansion associated with different patient groups (age, gender, stage of disease at diagnosis, staging as per the International Neuroblastoma Risk Group (INRG) Classification System, MYC-N amplification, and presence of segmental chromosomal aberrations). 2 patients had particularly good TILs expansion using both the initial and rapid expansion technique. Both patients were female, had L2 stage disease and had no tumor NMYC amplification or segmental chromosomal abnormalities. Both patients had received chemotherapy prior to attainment of the biopsy sample. 1 patient had particularly aggressive disease with recurrent localized disease and had received CAR-T cell therapy prior to biopsy samples being obtained.

### The expanded TILs population is a mixture of CD4^+^ and CD8^+^ lymphocytes with predominantly effector memory phenotype

**Fig 2:**
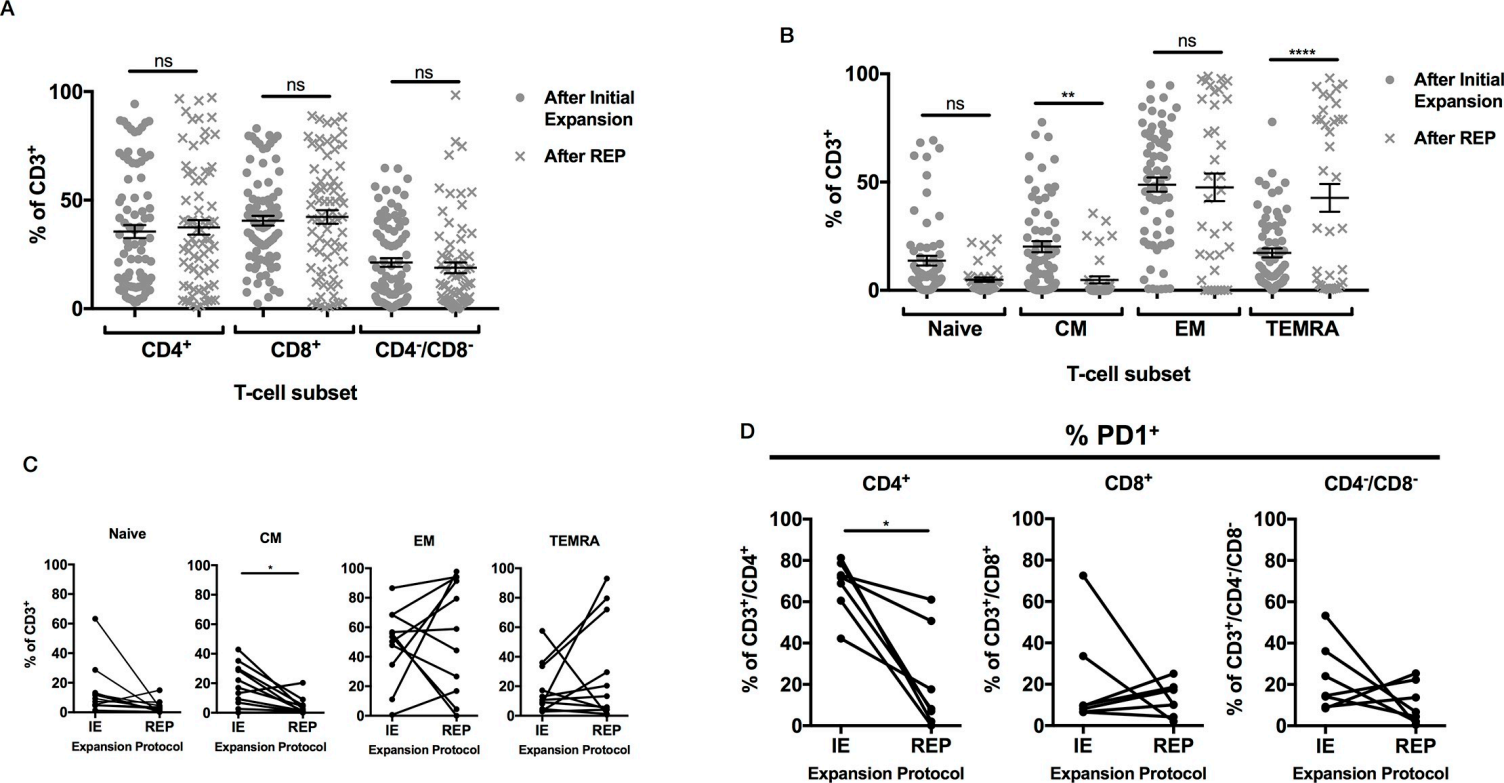
Phenotypic analysis of TILs after initial and REPs. (A) T-cell subsets shown as % of CD3^+^ cells (n = 75). Statistical comparison by one-way Analysis of Variance (ANOVA) with Sidak’s correction for multiple comparisons. (B) Memory phenotype of T-cells after initial expansion and after the REP (n = 63) using the following criteria: Naïve CD62L^+^/CD45RA^+^/CD27^+^, CM CD62L^+^/CD45RA^-^/CD27^+^, EM CD62L^-^/CD45RA^-^/CD27^-^, TEMRA CD62L^-^/CD45RA^+^/CD27^-^. There was a significant reduction in the proportion of CM cells (p = 0.007) and a significant increase in the proportion of TEMRA cells (p = <0.0001). Statistical comparison by one-way ANOVA with Sidak’s correction for multiple comparisons. (C) Memory phenotype for paired TIL samples (n = 12) after initial expansion and then following REP. Proportions of CM cells reduced significantly after REP (p = 0.038). Statistical comparison by paired one-way ANOVA with Sidak’s correction for multiple comparisons. (D) PD1 status of paired CD4^+^. CD8^+^ and CD4^-^/CD8^-^ T-cells after initial expansion and then following REP (n = 7). The percentage of CD4^+^ cells expressing PD1 fell significantly after REP (p = 0.015). Statistical comparison by paired one-way ANOVA with Sidak’s correction for multiple comparisons.

Analyzing by flow cytometry, after 14 days of initial expansion, 84% of gated cells (SD 11.4) were CD3 positive, of which there were CD4^+^ (35.5±3.0%), CD8^+^ (40.6±2.2%), and CD4^-^/CD8^-^ (21.3±2.02%) subsets. After REP, the distributions of T-cell subsets did not change significantly (37.5±3.3% vs. 42.3±3.1% vs. 18.9±2.5% respectively) (Fig 2A).

Memory phenotype was evaluated using CD45RA combined with either CD27 or CD62L. TILs were divided in naïve (CD62L^+^CD27+CD45RA+), Central Memory (CM) (CD62L^+^CD27+CD45RA-), Effector Memory (EM) (CD62L^-^CD27-CD45RA-) and Effector Memory/CD45RA (TEMRA) (CD62L^-^CD27- CD45RA+). At the end of initial expansion EM was the predominant phenotype (48.8±3.3%), followed by CM T cells (20.2±2.6%) (Fig 2B). However following REP there had been a significant reduction in the CM compartment to 4.8±1.6%, (p = 0.007) and corresponding increase in TEMRA to 42.7±6.4% (p<0.0001) with other populations remaining essentially unchanged (Fig 2B). In order to determine whether these changes were consistent between donors, 12 samples with data sets at initial expansion and post REP were compared. The reduction in the CM compartment was observed here (p = 0.037) but no significant differences were seen in the other subsets. There was a general pattern of reduction in less differentiated subsets and increases in more differentiated subsets (Fig 2C).

Programmed cell death protein-1 (PD1) staining was evaluated as a marker of activated/exhausted cells. After rapid expansion, the proportion of PD1 expressing CD4^+^ cells fell significantly in CD4 T cells (p = 0.014) and showed slight but non-significant changes in CD8^+^ and CD4^-^/CD8^-^ subsets (Fig 2D). In conclusion expanded TILs were heterogeneous in phenotype between patients, became more differentiated but expressed modest levels of exhaustion markers. There was a surprising high proportion of CD3 cells staining negatively for both CD4 and CD8.

### High numbers of γδ T cells, mostly Vδ1 and non-Vδ1Vδ2, are found within expanded TILs from neuroblastoma

**Fig 3:**
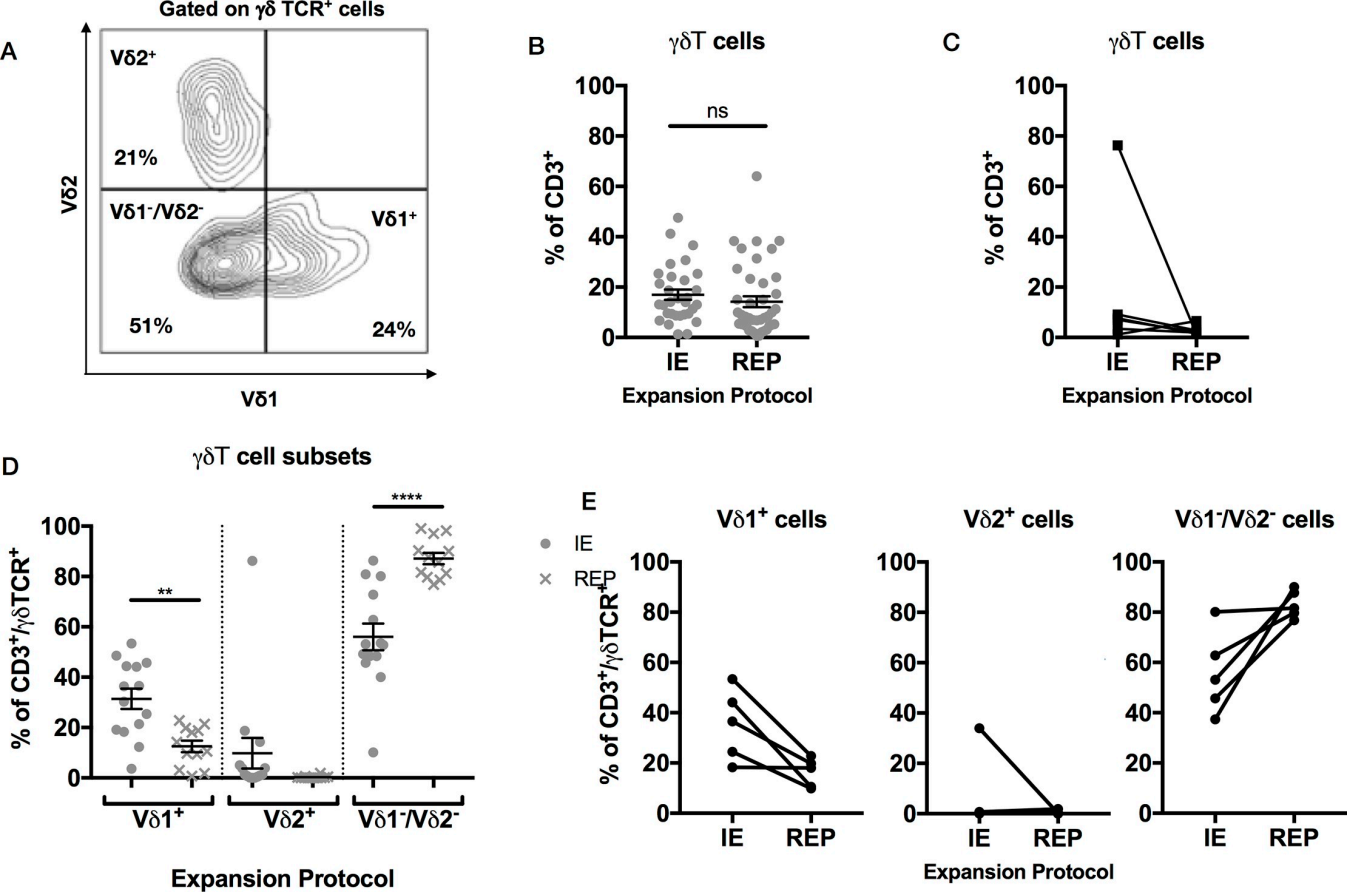
Innate lymphocyte subtype analysis within TILs population. (A) Gating strategy for identification of γδT cells subtypes after gating on CD3 and γδTCR positive cells. This representative example is from a TIL sample following initial 14-day expansion. (B) γδT cell numbers expressed as % of CD3^+^ cells in TILs following initial expansion (n = 31) and then after REP (n = 41): statistical analysis by unpaired t-test. (C) γδT cell numbers expressed as % of CD3^+^ cells in 6 paired TIL samples following initial expansion and then after REP. (D) γδT cell subset distributions in the TILs population defined as in (A) following initial expansion (n = 14) and then after REP (n = 12). Vδ1^+^ cell proportions fell significantly (p = 0.006) and Vδ1^-^/Vδ2^-^ proportions rose significantly after REP (p = <0.0001): statistical comparison by one-way ANOVA with Sidak’s correction for multiple comparisons. (E) γδT cell subset distributions in 5 paired TILs populations defined as in (A) following initial expansion and then after REP.

As we found a high proportion of CD4^-^/CD8^-^ T cells within the CD3^+^ TILs population, we hypothesized that these cells could be innate lymphocytes like Natural Killer-T (NKT) or γδ T cells, the presence of which has been associated with positive outcome in solid tumors [26]. TILs were defined as γδ T cells if they stained positively with anti-CD3 and anti-γδ TCR antibodies or were CD3+/αβTCR-. On the basis of staining with two delta chain specific antibodies, γδT cells were further subdivided into Vδ1^+^, Vδ2^+^ and Vδ1^-^/Vδ2^-^ subsets (Fig 3A). At the end of the initial expansion, 17±2.0% of the CD3^+^ TILs population were γδ T cells (n = 31) and after REP, the percentage was not significantly altered (14.2±2.2%, p = 0.36, n = 41) (Fig 3B). Moreover, analysis of samples where there was matched pre and post rapid expansion data (n = 6) confirmed lack of significant change in γδ T cell percentage (Fig 3C). Interestingly however we did observe a shift in the γδT cell repertoire within the γδ T cell compartment, with significant reduction in Vδ1^+^ (31.04±4.0% to 12.5±2.3%, p = 0.006) and increases in Vδ1^-^/Vδ2^-^ subsets (56±5.3% to 87.1±2.3%, p = <0.0001). The proportion of Vδ2^+^ γδT cells was low and fell even further though this did not reach statistical significance (9.8±6% to 0.28%0.5) (Fig 3D). Sample-linkage experiments demonstrated that the observed pattern of changes in the γδT cell repertoire was consistent between donors (Fig 3E). As the percentage of γδ T cells within the TILs population is higher compared to peripheral blood, which has 2–5% γδ T cells [27] and where the most common subset is Vδ2^+^, we reasoned that that they might play an important role in neuroblastoma.

### Expanded neuroblastoma TILs contain high numbers of NKT cells

**Fig 4:**
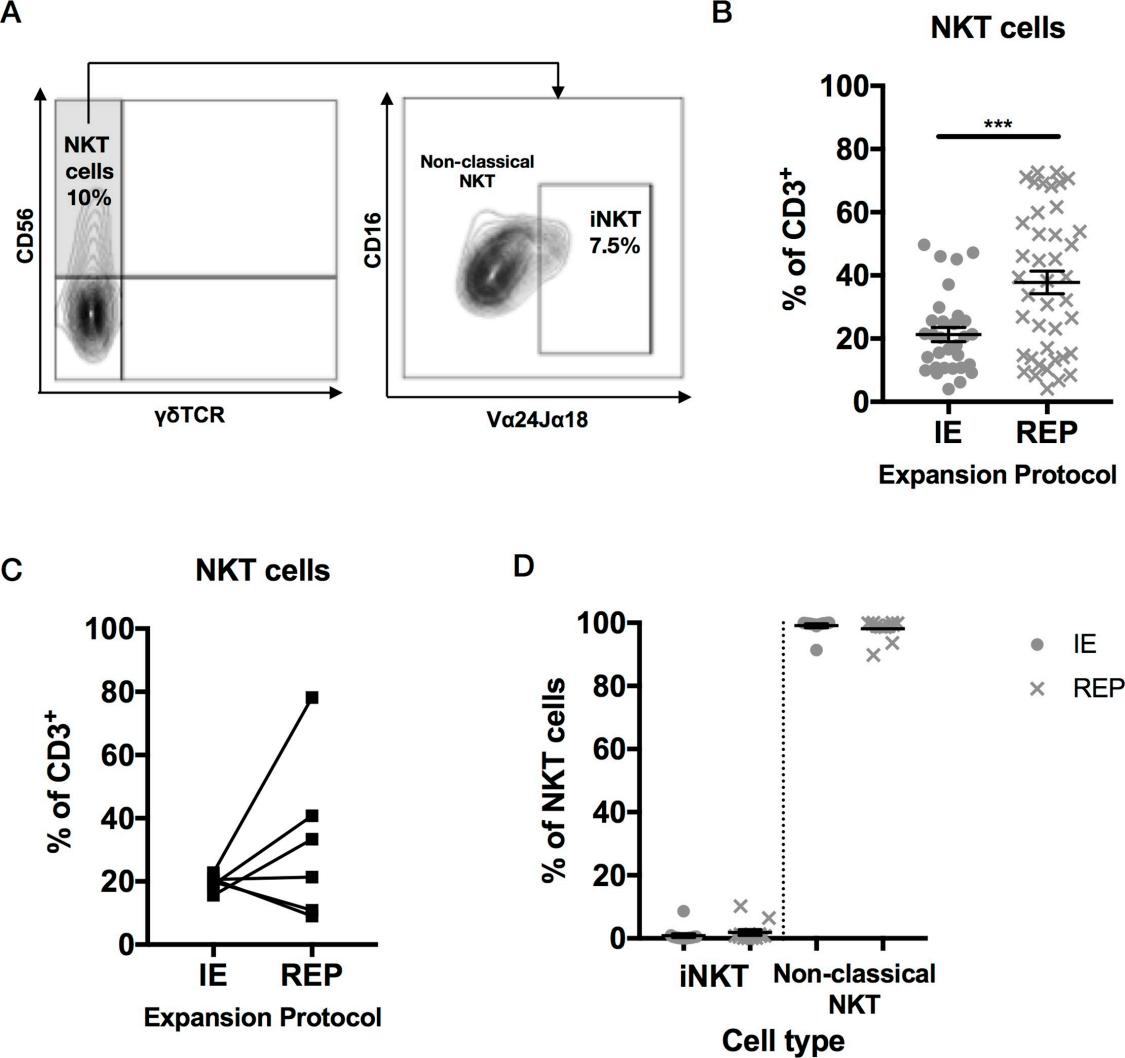
NKT subtype analysis within TILs population. Multiple TIL cultures were analyzed for each case after the 14 days of initial expansion (n = 31) and after REP (n = 41). (A) Gating strategy for NKT cell analysis. NKT cells are defined as CD3^+^/γδTCR^-^/CD56^+^. Invariant NKT (iNKT) are defined as CD3^+^/γδTCR^-^/CD56^+^/Vα24Jα18^+^ and non-classical NKT cells are defined as CD3^+^/γδTCR^-^/CD56^+^/Vα24Jα18^-^. Data shown from a representative TIL sample following initial expansion (14 days). (B) NKT cell numbers in TILs expressed as proportion on CD3^+^ cells following initial expansion (n = 31) and following REP (n = 41). NKT cell proportions rose significantly following REP (p = 0.0006 by unpaired t-test). (C) NKT cell numbers in TILs expressed as proportion on CD3^+^ cells following initial expansion and following REP in 5 paired samples. (D) NKT subsets within the TILs following initial expansion (n = 14) and following rapid expansion (n = 12). The majority of NKT cells fell in the CD3^+^/γδTCR^-^/CD56^+^/Vα24Jα18^-^ non-classical NKT cell gate, and the distribution of NKT cell subsets was not affected by the REP.

Another subgroup of innate lymphocytes is the natural killer T cells (NKT), which are positive for T cell markers such as CD3 but also positive for NK marker CD56. Defining NKT cells as CD3+CD56+γδTCR- (Fig 4A), at the end of initial expansion, 21.3±2.3% of CD3+ cells (n = 31 tumor fragments) had this phenotype compared with 37.8±3.6% following rapid expansion (n = 41 tumor fragments) representing a significant increase in NKT cell abundance (p = 0.0006 by unpaired t-test, Fig 4B). There was substantial donor to donor variability in NKT cell expansion dynamic between initial and rapid expansion when samples were linked (n = 5) (Fig 4C).

The NKT cell percentage within the TILs population was much higher than in blood, which contains average 4% NKT [28], suggesting that this cell type might play a role in neuroblastoma. NKT can be divided in two types: invariant, or type 1 NKT cells (iNKT), which express a highly restricted TCR composed by Vα24-Jα18/Vβ11, and non-classical, or type 2 NKT, which present a different CD1d-restricted TCR. To differentiate them, TILs were stained with the Vα24-Jα18 antibody and analyzed by flow cytometry (Fig 4A). Whilst in normal donor PBMC using this staining strategy we found the typical iNKT proportions in blood (not shown), after initial expansion, NKT cells in the TILs population were overwhelmingly non-classical NKT (99.1% ± 0.59); Vα24-Jα18^+^ iNKT only accounted for the 0.9% ± 0.59 of the total NKT population. At the end of REP, 1.86% ± 0.91 of the NKT population were iNKT cells and 98.2±0.9% were non-classical NKT cells (Fig 4D). Hence in our study expanded neuroblastoma TILs comprised a high proportion of the rare cell type of CD3+ cells expressing CD56 and lacking expression of Vα24-Jα18/Vβ11 TCR, consistent with the phenotype of non-classical type 2 NKT cells.

### Expanded TILs from neuroblastoma are largely non-reactive to autologous tumor cells but retain cytokinesis towards tumor cells

**Fig 5:**
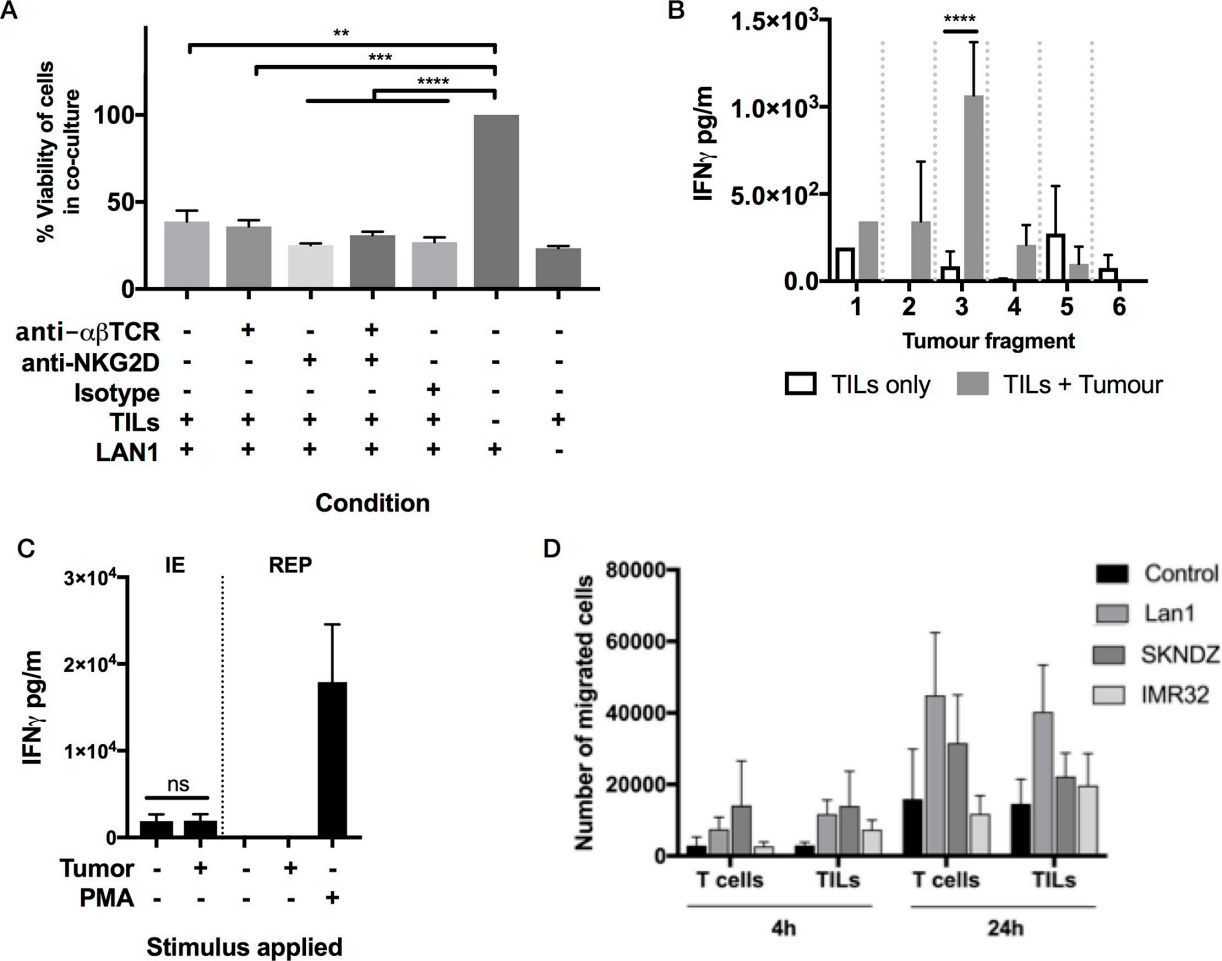
Functional potential of neuroblastoma expanded TILs. (A) LAN1 neuroblastoma cells were co-cultured overnight with TILs and cell viability was assessed using an MTT assay. Presence of TILs significantly reduced viability of LAN1 cells in a manner which could not be blocked using TCR or Natural Killer Group 2D (NKG2D) blocking antibodies. (B) Example of a donor whose TILs were able to mount an IFNγ response against 1/6 autologous tumor fragments. In this case, IFNγ production by TILs was significantly increased in the presence of a 1-2mm tumor fragment (p = < 0.0001 by paired 2-way ANOVA with Sidak’s correction). Error bars are ±SEM of technical replicates (n = 1–3) for each tumor fragment/TIL co-culture. (C) IFNγ secreted by TILs in the absence or presence of autologous neuroblastoma tumors after initial expansion (n = 24) or after REP (n = 6). Stimulation with PMA is used as a positive control to demonstrate the capacity to mount an IFNγ response. (D) Migration of T-cells and TILs towards neuroblastoma cell lines (Lan1, SKNDZ and IMR32) at two different time points (4 and 24 h). T-cells or TILs were placed in the top chamber of a trans-well and target cells were placed in the bottom. At 4h, TILs: n = 3 and T cells: n = 2. At 24h, TILs: n = 8 and T cells: n = 3. Data represented as mean ± SEM.

To evaluate innate anti-neuroblastoma responses, we cultured day 14 initial expansion TILs with allogeneic LAN1 neuroblastoma cells. Following overnight culture, an MTT assay showed a significant decrease in target cell viability consistent with innate cytotoxicity (Fig 5A). Using Interferon-gamma (IFNγ) production as a read out of tumor-specific recognition and signaling we demonstrated that some TIL cultures were able to mount a response against a 1-2mm fragment of autologous tumor following 24h co-culture whilst other co-cultures were non-reactive (Fig 5B). Tumor fragments used were small and it was not possible to quantify tumor cell content. Therefore, lack of reactivity in some cultures might be related to lack of access to the tumor cells or insufficient tumor cell content. To test whether expanded TILs had lost capacity to produce interferon gamma as an explanation of non-reactivity to autologous tumor, we found that initial expansion TILs had spontaneous IFNγ release, which when averaged over multiple donors and experiments was not increased on addition of autologous tumor. Following REP, spontaneous IFNγ production was completely abolished (Fig 5C). However, this was not due to the inability of the post REP TILs to produce the cytokine, as demonstrated by the robust response to phorbol myristate acetate (PMA) stimulus.

To evaluate the migration properties of 8 post REP TILs in comparison with freshly isolated T cells from healthy donors, a transwell migration assay against three different neuroblastoma cell lines was used. Averaged over the 8 representative TILs, all three cell lines promoted migration and no difference was observed between blood-derived T cells and expanded TILs (Fig 5D). Therefore, expanded TILs post REP retain capacity to produce interferon gamma if suitably activated, and retain the capacity of freshly isolated T cells to migrate towards neuroblastoma cell lines.

### GD2-specific CAR-TILs show tumor reactivity against GD2^+^ cell lines

Having demonstrated that expanded TILs from neuroblastoma retain capacity for IFNγ responses given appropriate stimulus and having shown they have *in vitro* migration properties towards neuroblastoma cells, we hypothesized that a chimeric antigen receptor (CAR) could be used to augment tumor-specific cytotoxicity in a cell population with natural tumor residency. TILs were transduced between day 8 and day 10 of REP with a GD2-CAR construct (GD2-(ζ) containing the Single chain variable fragment (ScFv) huK666, a humanized form of the murine anti-GD2 antibody muK666 [29].

**Fig 6:**
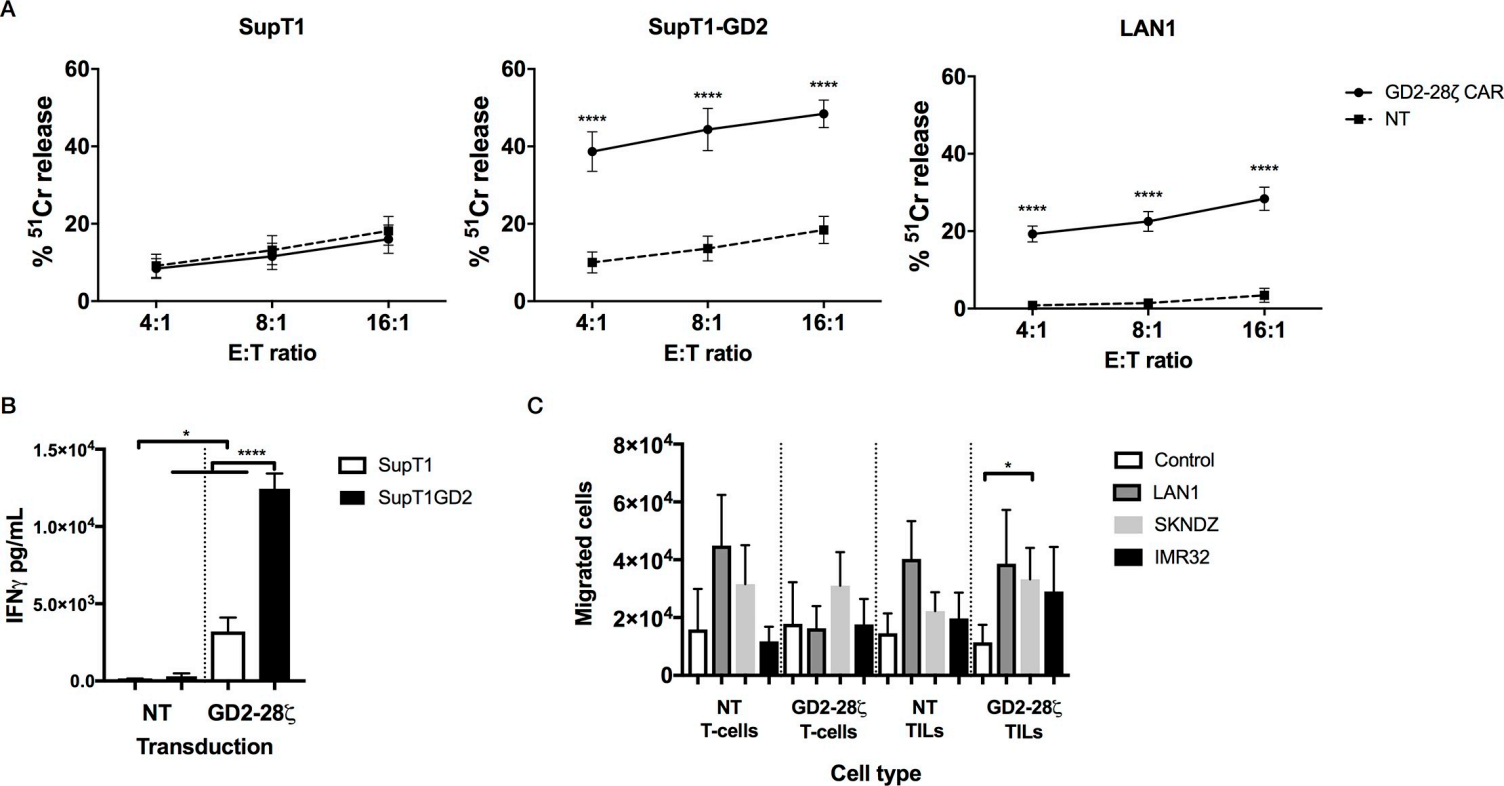
Functional analysis of chimeric antigen receptor expressing TILs. TILs were transduced to express an anti GD2-28ζ CAR with a mean transduction efficiency of 16.2±2.8%. (A) Killing of GD2^-^ SupT1, GD2^+^ SupT1-GD2 and GD2^+^ LAN1 neuroblastoma cells by transduced and non-transduced (NT) TILs (n = 16). Killing of SupT1-GD2 and LAN1 was significantly enhanced by the presence of GD2-28ζ (p = <0.0001). Statistical analysis by 2-way ANOVA with Sidak’s correction for multiple comparisons. (B) IFNγ release by non-transduced and GD2-28ζ transduced TILs in response to SupT1 or GD2-SupT1 (n = 8). Presence of the GD2-28ζ CAR increases background IFNγ production (p = 0.011) but the presence of GD2^+^ SupT1-GD2 leads to much greater IFNγ release (p = <0.0001). Statistical analysis by one-way ANOVA with Sidak’s correction for multiple comparisons. (C) Transduction with GD2-28ζ does not reduce the migratory capacity of TILs towards neuroblastoma cell lines, as determined using a 24h trans-well assay with target cells in the bottom chamber and effectors in the top chamber.

Transduction efficiency of 16.2±2.8% was achieved over 35 replicates. For assessing cytotoxicity, the percentage of cells expressing CAR was standardized for each assay through addition on non-transduced cells (CAR expression determined via co-expressed CD34 selection marker). CAR-TIL demonstrated a significantly higher levels of antigen specific cytotoxicity (p = <0.0001 at all effector:target ratios by 2-way ANOVA with Sidak’s correction) in 4-hour ^51^Cr release killing assays against GD2^+^ LAN-1 neuroblastoma cells and SupT1 engineered to express GD2 (SupT1-GD2). No enhancement in killing of GD2^neg^ SupT1 was seen (Fig 6A). Moreover, in parallel co-culture experiments, GD2-CAR TILs produced significantly higher IFNγ levels in an antigen-specific manner than non-transduced controls (p = <0.0001). There was however a small but still significant (p = 0.011) increase in background IFNγ production by CAR-TILs compared to non-transduced controls, consistent with a degree of tonic CAR signaling (Fig 6B).

Migration properties of CAR TILs were next evaluated with a transwell migration assays. The number of migrated cells was higher after 24h compared to 4h. At 24 hours there was an increase in mean migration toward neuroblastoma lines compared with controls, but no difference was observed between TILs and T cells derived from normal blood donors. Furthermore, no difference in migratory capacity could be attributed to the presence of the CAR in either T cells or TILs (Fig 6C). At 24h, the perceived enhanced migration of CAR TILs towards neuroblastoma lines reached significance for the SKNDZ line (p = 0.026) compared to control (Fig 6C). Therefore, there is no evidence that the presence of the CAR in expanded neuroblastoma TILs interferes with their *in vitro* migratory properties.

## Discussion

Adoptive T cell therapy (ACT) based on TILs has demonstrated high overall response rates and sustained regressions, almost exclusively in patients with metastatic melanoma [30]. In recent years, new data have pointed towards TILs therapy to further improve survival of patients with other solid malignancies [31,32].

Because of low HLA molecule expression [33] and low mutational load compared to melanoma, neuroblastoma has been considered to be a non-immunogenic tumor [34,35]. However, in 1968 blood lymphocytes from neuroblastoma patients were reported to inhibit the colony formation by both autologous and allogeneic tumor cells [36]. More recently studies have demonstrated the activity of both the innate and adaptive immune system in neuroblastoma. For example NK cell depletion in mice bearing allografted tumors from syngeneic TH-MYCN transgenic mice led to enhanced tumor growth [37] whilst other studies have shown the accumulation and tumor reactivity of CD3^+^CD8^+^ T cells within the neuroblastoma microenvironment [10,25].

To date, the characterization of TILs in neuroblastoma in terms of phenotype, frequency, specificity and response to expansion protocols *in vitro* is sparse, as compared with other tumor types such as melanoma. We therefore expanded and characterized TILs from 30 pediatric neuroblastoma samples. Three observations gave evidence of the potential of TILs as a new immunotherapy to treat neuroblastoma: TILs were readily isolated and expanded from neuroblastoma tissue; a relatively high percentage of innate lymphocytes was found within the TILs population; and TILs transduced with GD2-CAR showed specific killing activity.

Adoptive cell therapy (ACT) relies on both the quality and quantity of cells expanded from the original source. We showed in this study that TILs can expanded from 90% of the neuroblastoma cases, and clinically relevant numbers can be obtained after REP. Of note in our study relatively small tissue fragments were used whereas for a clinical study, the process could be scaled up potentially 10-fold or more. Since tumor samples with lower number of infiltrating TILs will take longer to expand to the same numbers, this ability to scale up will be important for clinical translation [38]. One speculative approach to overcome this limitation, would be pre-treatment with checkpoint inhibitors before surgical resection in order to enrich tumor tissue for the lymphocyte population desired. That would shorten the time between surgery and infusion through speedier expansion of a target cell dose for adoptive transfer. This has been previously shown in animal studies, in which anti-PD-L1 antibody treatment demonstrated a significant increase in T cell infiltration in colon cancer bearing mice, suggesting that PD-L1 blockade is an effective strategy to increase CD8^+^ T cell infiltration [39]. In a human setting, it has also been shown that anti-CTLA-4 antibody treatment prior to tumor resection is feasible and well-tolerated in patients with metastatic melanoma [40].

The analysis of TILs by flow cytometry revealed a mixed population of both helper and cytotoxic T cells. For ACT of TILs, skewing toward CD8^+^ vs. CD4^+^ T cells is thought to provide better clinical outcome, as cytotoxic T cells are more reactive against the tumor. For instance, a link between the number of administered CD8^+^ T cells and the objective tumor regression was observed in a phase II study in patients with melanoma [41]. On the other hand, the presence of other cell types in the TILs population, such as CD4^+^ T cells, is needed to induce clinical responses as it has been reported that, in a minority of patients, the transfer of a CD4^+^ T cell dominant TIL product leaded to dramatic clinical responses and even complete remission of their tumors [42]. Another important feature for ACT is the prevalence of “young TILs”, including naïve and CM T cells, over EM and TEMRA cells. Although EM cells have a higher cytotoxic activity and a greater capacity to secret pro-inflammatory cytokines such as IFNγ [43], naïve and CM T cells are less differentiated in terms of effector function and therefore have a better potential of self-renewal [44]. REP with high-doses of IL-2 and anti-CD3 antibody induces a more differentiated phenotype in T cells. To overcome that problem, further experiments with different cytokine cocktails, such as IL-12, could be performed in order to achieve similar T cell expansion but less differentiated TILs [45].

An unexpected finding in our results was the high percentage of double negative (for CD4 and CD8) lymphocytes within the TILs population from neuroblastoma, which suggested to us infiltration of innate lymphocytes, such as γδ T cells and NKT cells. The γδ TCR is relevant due to its capacity to recognize novel ligands that are not identified by the αβ TCR, thereby providing a crucial additional pathway of local immunosurveillance important for tumor defense. One of the methods that neuroblastoma uses to escape from the immune system is the downregulation of MHC class I molecules. γδ T cells could be a solution to this problem, as they are able to differentiate between healthy and cancer cells in an MHC-independent manner [46]. Also, a published study reported human γδ T cells to exert cytotoxicity towards neuroblastoma cells [47]. We predominantly found γδ T cells expressing the *Vδ1* and *non-Vδ1/Vδ2* genes within the TILs population, suggesting a tumor tropism for these cell types. The Vδ1 subset could be an optimal choice for anti-tumor immunotherapy as they have lower expression of PD1 as well as a less differentiated memory state after expansion compared to Vδ2 lymphocytes [48]. NKT cells also present a high anti-tumor potential in an MHC-independent manner. Previous studies reported a link between high infiltration of invariant NKT cells in neuroblastoma tissue and positive patient prognosis; however, we could not find a clear iNKT population within the neuroblastoma expanded TILs [49,50].

Finding that expanded TILs from neuroblastoma patients did not consistently exhibit any cytotoxic activity against autologous GD2^+^ cell lines was somewhat unexpected. There might be technical limitations to these experiments given the difficulty in assessing tumor cell content in the tiny fragments available for our co-cultures. Moreover, given the high number of innate-like lymphocytes within our TIL cultures it will be important for future studies to evaluate the cytotoxicity of these subpopulations against autologous tumor. This will potentially identify the infiltrating cells that are involved in tumor control, as well as prioritizing cell populations for targeted expansion.

In order to increase specificity against the desired target, we transduced TILs with a CAR. Direct genetic manipulation of TILs has been previously reported but only with the purpose of improving TIL survival and function. For instance, TILs transduced with a gene encoding a dominant-negative form of the TGF-β receptor increased resistance to inhibition mediated by TGF-β, an immunosuppressive cytokine produced by about 50% of melanoma cancers [51]. Moreover, another approach was to manipulate the cellular apoptotic machinery of TILs. Overexpression of antiapoptotic molecules, such as Bcl-xL or Bcl-2, promoted lymphocytes survival as well as improved anti-tumor efficacy [52]. Our strategy was to transduce TILs with a second-generation GD2 specific CAR, being evaluated in a clinical trial. We found that transduction of TILs with the GD2-CAR was possible and CAR TILs showed higher killing capacity against cell lines expressing GD2 compared to their non-transduced counterparts, indicating specific reactivity of CAR TILs against neuroblastoma.

In conclusion, our study demonstrates the potential use of expanded TILs in therapeutic application in pediatric neuroblastoma, and the potential of CAR TILs as a new therapy. The ability of CAR TILs to react to tumor antigen stimulation shows that this population has the capacity to elicit a tumor-specific response. Hence, ACT using CAR TILs is a therapeutic approach deserving of further study.

## Materials and methods

### Patients, samples and research ethics

30 samples derived from 24 patients with neuroblastoma were included in this study. All tumor samples were collected from patients at the Great Ormond Street Hospital following written informed consent. In the case of minors, consent was obtained from adults with parental responsibility, with written assent from the child being optional for older participants. Ethical approval for the study was obtained through the national United Kingdom research ethics application system of the UK Health Research Authority; reference 14/WM/1253 “Establishing primary cultures and cell lines from pediatric cancers”. Tumor tissue was disaggregated manually into 1–2 mm fragments in X-VIVO 15 media (BE02-060F, Lonza) supplemented with 5% heat-inactivated human AB serum (H3667, Sigma-Aldrich), 100IU/mL penicillin (P4333, Sigma-Aldrich) and 100 μg/mL streptomycin (P4333, Sigma-Aldrich).

### Cell lines

Cell lines used in this study were acquired from ATCC. These included 293T, Lan1, SupT1, SupT1GD2, IMR-32 and SKNDZ. 293T and SKNDZ cells were cultured in Iscove’s Modified Dulbecco’s Medium (IMDM, 12-722F, Lonza) supplemented with 10% heat-inactivated fetal calf serum (FCS, A3160802, Gibco), 1% penicillin/streptomycin (P4333, Sigma-Aldrich) and 1% L-glutamine (G7513, Sigma-Aldrich). SupT1, IMR-32 and Lan1 cells as above without L-glutamine and in RPMI 1640 (12-115Q, Lonza) (SupT1 and Lan1) or Eagle’s Minimum Essential Medium (12-137F, EMEM, Lonza) (IMR-32).

### Initial expansion of TILs

For the first 2 weeks, TILs were stimulated with Dynabeads Human T Activator CD3/CD28 (11131D, Life Technologies) in the presence of high dose IL-2 (Proleukin, Prometheus) as previously described(5). Briefly, one 1-2mm tissue fragment per well was placed in a 24-well plate in 2 mL of complete X-VIVO 15 media supplemented with 1x10^6^/mL CD3/CD28 Dynabeads and 3000 IU/mL IL-2, and then cultured at 37°C in 5% CO_2_. 3000 IU/mL IL-2 were added on alternate days. Cells were split to maintain a density of 1x10^6^ cells/ml. Negative control cultures lacked IL-2 and CD3/CD28 beads. After 7 days IL-2 was reduced to 1000 IU/mL. On attaining confluency cell were resuspended to obtain a more uniform distribution. After 14 days, the CD3/CD28 Dynabeads were removed with a Dynal MPC-S Magnet (A13346, Thermo Fisher) according to manufacturer instructions, and cells counted. If TILs did not expand after 14 days, the initial expansion was extended or TIL cultures were discarded.

### Rapid expansion of TILs

0.25x10^6^ expanded TILs were placed in 75cm^2^ culture flasks with 25 mL of complete X-VIVO 15 media, 1000 IU/mL IL-2, 30 ng/mL anti-CD3 monoclonal antibody (OKT-3, 130-093-387, Sigma-Aldrich), and allogenic irradiated (50Gy) PBMC as feeder cells at a ratio of 1:200 (TIL:feeders). Irradiated PBMCs with complete X-VIVO 15 media were used as the control. 1000 IU/mL IL-2 were added to the rapid expansion on alternate days. After 14 days of rapid expansion, TILs were collected and counted manually.

### Construction of retroviral constructs to express chimeric antigen receptors

The retroviral vector SFGmR.RQR8-2A-aGD2huK666-HCH2CH3pvaa337-CD28Z (referred to as GD2-CAR hereafter) encodes a CAR of which the ectodomain comprises the huK666 ScFv (antibody against human GD2) (29), a spacer derived from the human IgG4 CH2-CH3, and CD28 and CD3ζ intracellular signaling domains. The marker/suicide gene RQR8 was co-expressed with the GD2-CAR using a foot and mouth virus self-cleaving 2A peptide allowing detection of transduced cells by staining with QBEND10 anti-CD34 antibody as previously described [53]. The retroviral vector used was the oncoretroviral vector SFG[54], pseudotyped with an RD114 envelope.

### Transduction of T cells

Gamma retroviral particles were generated as previously described (29). For transduction, non-tissue-coated 24-well plates were coated with 0.5 mL/well retronectin (T100B, Takara Bio) at a concentration of 1 mg/mL overnight at 4°C. The next day, TILs between day 8 and 10 of REP were counted manually and resuspended to a concentration of 0.7x10^6^ cells/mL in complete X-VIVO 15 media supplemented with 1000 IU/mL IL-2. Retronectin was removed from the plate and 0.5 mL of activated T cells and 1.5 mL of viral supernatant were added to each well. The plate was centrifuged at 1000x g for 40 min to effect transduction. 3 days later TILs were harvested and pooled together in a 75cm^2^ flask with complete X-VIVO 15 media and 1000 IU/mL IL-2. Non-transduced control wells were filled with media instead of retroviral supernatant. Transduction efficiency was determined by flow cytometry 5 days after transduction. The cells were stained with APC-conjugated anti-CD34 antibody (clone QBEnd10, FAB7227A, R&D Systems), FITC-conjugated anti-CD3 antibody (clone UCHT1, 11-0038-41, eBioscience) and PerCP AffiniPure Goat Anti-Human IgG (Fcɣ fragment specific, 109-125-098, Jackson ImmunoResearch).

### Flow cytometry for phenotype analysis

Cells were collected and washed once with FACS buffer prior to extracellular staining. To prevent competitive binding of Vδ2 antibody with pan-ɣδTCR antibody, cells were stained with PE-Cy7- pan-ɣδTCR antibody (clone B1, 331221, BioLegend) or BV395 pan-ɣδTCR antibody (clone B1, 566215, BD Bioscience) for 15 mins at 4°C in the dark before adding all the other antibodies. Extracellular staining was performed for 30 min at 4°C with the following fluorescent-labelled antibodies diluted with FACS buffer in a total volume of 100 μL: BV421 PD1 (CD279, clone EH12.2H7, 329919, BioLegend), BV605 CD27 (clone O323, 302829, BioLegend), APC-conjugated CD34 (clone QBEnd10, FAB7227A, R&D Systems), APC-Cy7-conjugated CD3 (clone UCHT1, 300425, BioLegend), BV711 CD8 (clone RPA-T8, 301043, BioLegend), BV650 CD4 (clone OKT4, 317435, BioLegend), PE-Cy7-conjugated CD45RA (clone HI100, 304125, BioLegend), FITC-conjugated Vδ1 (clone TS8.2, TCR2730, ThermoScientific), APC-conjugated αβ TCR (clone IP26, 306717, BioLegend), BV758 CD3 (clone OKT3, 317329, BioLegend), APC-Cy7-conjugated CD56 (clone 5.1H11, 362511, BioLegend), BV421 Vα24-Jα18 (clone 6B11, 342915, BioLegend), PE-conjugated Vδ2 (clone REA711, 130-111-126, Miltenyi Biotec), FITC-conjugated CD45 (clone HI30, 555482, BD Bioscience) and BUV496 CD16 (clone 3G8, 564654, BD Bioscience). Flow cytometry data were acquired on BD LSRII flow cytometer (BD Bioscience). Compensation was carried out using OneComp eBeads (01-1111-41, eBioscience) stained with single colors. To determine the placement of the gates, appropriate fluorescence minus one (FMO) and unstained controls were used. Results were analyzed using FACSDiva software (Beckton and Dickinson) and FlowJo software.

### Cytotoxicity assay

*In vitro* cytotoxic activity of transduced and non-transduced TILs was evaluated in a standard 4 h ^51^Cr-release assay at 4 different effector:target (E:T) ratios. The assays were performed in triplicate and the mean percentage of specific lysis of triplicate wells was calculated according to the formula: 100 x (experimental release-spontaneous release) / (maximum release-spontaneous release). In some experiments, cytotoxicity was determined using the same formula but based on remaining viable target cells (after removing effectors) estimated using an MTT (3-(4,5-dimethylthiazol-2-yl)-2,5-diphenyltetrazolium bromide, Abcam) assay according to manufacturer’s instruction.

### Co-culture of TILs with tumor cells

Autologous tumor cells for co-culture assays were either fragments of manually disaggregated tumor or short-term cultures grown in stem cell media [55]. For each co-culture, TILs were seeded at 10,000 cells/well of a round bottom 96-well plate and cultured with targets at 1:1 ratio when target cell count was possible. For some experiments with tumor fragments and neurospheres, cell number needed to be estimated. As a positive control, effectors were stimulated with lyophilized phorbol 12-myristate 13-acetate (PMA, 20 ng/ml, sc-3576, Santa Cruz Biotech) and Ionomycin (1μg/ml, 10004974, Cayman Chemical). As a readout for tumor reactivity, IFNγ production was measured by ELISA from the culture supernatant using ELISA MAX Deluxe Set was used according to manufacturer’s protocol (430104, BioLegend) according to manufacturer’s instructions.

### Transwell migration assay

Following transduction, migratory potential of T cells was analyzed in a chemotaxis assay using 24-well culture plates carrying polycarbonate membrane-coated transwell permeable inserts of 5 μm pore size (3421, Costar Transwell, Corning). 2x10^5^ TILs or control T cells were seeded in the upper wells, and lower wells contained Lan1, SKNDZ or IMR-32 overnight cultures of 0.5x10^6^ tumor cells or 600 μL of complete media negative control. Cell migration was measured after 4 h or 24 h at 37°C and 5% CO_2_ using counting Beads (424902, BioLegend).

### Statistical analysis

Prism 7 software (GraphPad) was used to analyze data by one-way ANOVA test (when comparing data in three or more groups with one variable) or two-tailed student’s t-test (when comparing data only in two groups). Differences were considered statistically significant at *p ≤ 0.05, **p ≤ 0.01, ***p ≤ 0.001 and ***p ≤ 0.0001. Where numerical results are displayed in the text they are displayed as mean ± SEM.
